# Hemophagocytic lymphohistiocytosis following pembrolizumab and bevacizumab combination therapy for cervical cancer: a case report and systematic review

**DOI:** 10.1186/s12877-023-04625-3

**Published:** 2024-01-08

**Authors:** Chongya Zhai, Xuanhong Jin, Liangkun You, Na Yan, Jie Dong, Sai Qiao, Yuhong Zhong, Yu Zheng, Hongming Pan

**Affiliations:** 1 Department of Medical Oncology, Sir Run Run Shaw Hospital, College of Medicine, Zhejiang University, 3 East Qingchun Road, Hangzhou, 310000 Zhejiang Province People’s Republic of China; 2 Key Laboratory of Digital Technology in Medical Diagnostics of Zhejiang Province, Dian Diagnostics Group Co.,Ltd, Hangzhou, China; 3 Department of Medical Oncology, Shaoxing Campus, Sir Run Run Shaw Hospital, College of Medicine, Zhejiang University, Shaoxing, China; 4 Key Laboratory of Precision Medicine in Diagnosis and Monitoring Research of Zhejiang Province, Sir Run Run Shaw Hospital, College of Medicine, Zhejiang University, Hangzhou, China

**Keywords:** Hemophagocytic lymphohistiocytosis, Pembrolizumab, Immune-related adverse event

## Abstract

**Background:**

Programmed cell death protein 1 (PD-1) checkpoint inhibitors such as pembrolizumab are novel therapeutics used to treat various advanced malignancies. Immune-related adverse events are common, among the most serious of these toxicities is hemophagocytic lymphohistiocytosis (HLH), which is a life-threatening disorder of unbridled immune activation but has not been properly established.

**Methods:**

We have procured the first case of hemophagocytic lymphohistiocytosis as an aftermath of treatment with pembrolizumab from the Sir Run Run Shaw Hospital, Zhejiang University, China. In a pursuit to enhance the understanding of this condition, a comprehensive systematic review was performed encompassing all reported instances of ICI-associated Hemophagocytic lymphohistiocytosis within the realms of PubMed and Embase databases.

**Results:**

We detail the recovery of a cervical cancer patient with a history of psoriasis who developed HLH after combined pembrolizumab and bevacizumab treatment. Remarkably, tumor lesions exhibited substantial and sustained regression. From an analysis of 52 identified Immune Checkpoint Inhibitor (ICI)-related HLH cases, we discovered that HLH often occurred within the first two treatment cycles and approximately 20% of these patients had a history of autoimmune-related diseases. Despite a 15% mortality rate, the majority of patients experienced positive outcomes. Notably, in instances of recovery from HLH, 80% showed positive tumor outcomes. Even after discontinuation of ICI treatment, tumor control persisted in some cases.

**Conclusion:**

We identified the first case of HLH caused by ICI treatment in cervical cancer and summarized the possible occurrence factors of these cases, the treatment outcomes of HLH, and the impact on tumor outcomes.

**Supplementary Information:**

The online version contains supplementary material available at 10.1186/s12877-023-04625-3.

## Background

Cancer immunotherapy using immune-checkpoint inhibitors (ICIs) has emerged as a cornerstone treatment for various cancers. However, by eliminating the physiological inhibitory control that moderates T-cell activation, ICIs may cause T-cell hyperactivation and immune-related adverse events (irAEs) [1], one rare but serious irAE is hemophagocytic lymphohistiocytosis (HLH). HLH is a severe condition caused by immune activation and dysregulation, potentially leading to excessive pro-inflammatory cytokine secretion, rapid tissue destruction, multi-organ failure, and death [2]. Although the incidence of ICI-associated HLH is very low, with the World Health Organization's VigiBase pharmacovigilance database reporting only 5.7% of all HLH cases as potentially ICI-associated [3], the Society for Immunotherapy of Cancer (SITC) includes HLH in lethal irAE but does not provide specific treatment recommendations [4].

In the phase I/II CheckMate 358 trial (NCT02488759) [5] and KEYNOTE-158 (NCT02628067) [6], large clinical trials confirming the safety of ICIs in cervical cancer, ICIs have been widely used for cervical cancer treatment. However, no serious hematological adverse reactions due to ICIs in cervical cancer have been identified recently, and we report the first case of HLH attributed to ICIs in cervical cancer. This finding has not been previously documented in the literature or pharmacovigilance databases.

To better understand the biomarkers of ICI-associated HLH, clinical features, treatment strategies, characteristics of deceased cases, and the potential impact of HLH on tumor outcomes, we gathered all cases that met the HLH-2004 [7] or HScore > 169 criteria [8]. (Supplemental data 1) for ICI-related HLH. In total, we analyzed 52 confirmed ICI-related HLH cases by comparing our collection with data from two WHO pharmacovigilance databases. Our review revealed an increasing occurrence of ICI-related HLH in various cancer types and ICI classifications, a high number of cases with a history of autoimmune disease in HLH occurrence, autoimmune encephalitis as a significant cause of death, and the potential for an exceptional oncologic response to treatment if recovery from HLH is achieved.

## Methods

### Case report

The clinical case of HLH was encountered by the authors in their clinical practice at Sir Run Run Shaw Hospital, Zhejiang University, China. Clinical data were gathered through review of the electronic patient journal. Data were visualized using GraphPad Prism V.9.4.0.

### Systematic review

We conducted a systematic search using medical subject headings (MESH) in PubMed and Embase databases up to April 2023 to explore the keywords "Hemophagocytic Lymphohistiocytosis" and "immune checkpoint inhibitors". Search strategy free words and grid terms are as follows: (“Immune checkpoint inhibitor”OR “anti-PD1”OR“anti-PD-L1”OR “anti-CTLA4”“avelumab” OR “atezolizumab” OR “cemiplimab” OR “durvalumab”, OR “ipilimumab” OR “nivolumab” OR “pembrolizumab”OR “camrelizumab”) AND (“Hemophagocytic Lymphohistiocytosis” OR “HLH” OR “Macrophage Activation Syndrome”OR “lymphohistiocytic syndrome”OR“hemophagocytic syndrome”). Through manual retrieval of the references included in this study, other relevant studies that were not found in the retrieval process in databases were found shows the literature acquisition flow chart for the meta-analysis. (Supplemental data 2) Inclusion criteria for abstract review were assessed independently by two reviewers (XHJ and CYZ) and defined as follows:(1) report on HLH or related systemic syndromes on human patients with malignant tumors. (2) report use of ICI as the primary therapeutic agent before the onset of HLH. (3) case series or case reports, that is, no randomized controlled trial. (4) meet Hscore > 169 or meet at least five of the eight criteria in HLH-2004.(The specific search formula and flowchart are shown in Supplemental data 2).

## Results

### Case report

We report the case of a 73-year-old Chinese woman with a medical history of psoriasis. Pathological examination suggested moderately differentiated squamous cell carcinoma, and a comprehensive PET-CT scan revealed a 72*52 mm soft tissue mass in the cervix with lesions invading the uterus upwards and the upper third of the vagina, increased FDG uptake with a maximum SUV of 21.33, several mass lymph nodes in the mediastinum, right pulmonary hilar, and pelvis, increased FDG uptake with a maximum SUV of 15.44. Leading to a diagnosis of stage IVB cervical squamous carcinoma. NGS testing indicated PD-L1: TPS = 71%-80%, Microsatellite stable, TMB = 8.96Muts/Mb, and mutations in AKT1, KMT2C and NF1. The patient, initially undergoing one course of paclitaxel and cisplatin treatment, transitioned to pembrolizumab and bevacizumab therapy due to fatigue.

**Fig. 1:**
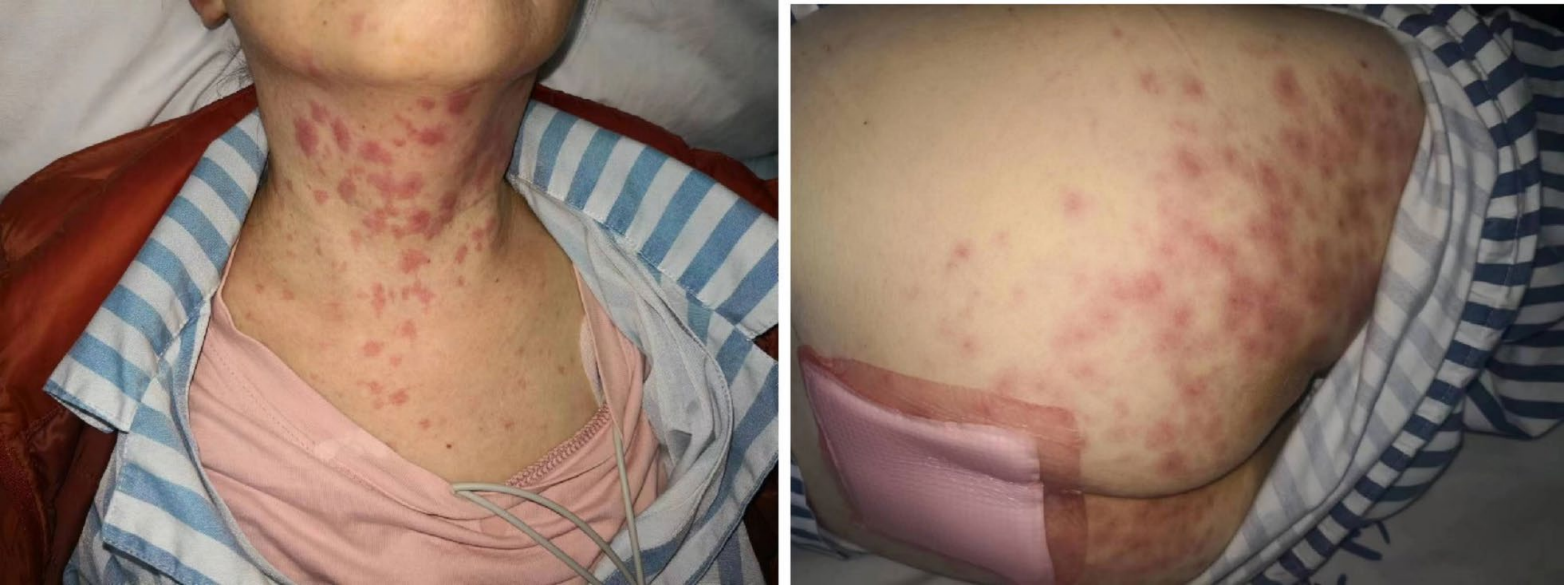
Physical findings. Macular rash observed on the bilateralis buttock, thighs and cervico-thoracic region

Seven days after the first cycle, the patient was readmitted to the hospital with fever, dry mouth, and right iliac pain, with a maximum temperature of 38.5 degrees Celsius. Physical examination showed no congestion or swelling in the throat, slight breathing sounds in both lungs, no obvious dry or wet rales and no obvious tenderness or rebound pain in the entire abdomen. Following abdominal CT scans, no sign of active inflammatory lesion was observed. Results from laboratory blood tests revealed that leukocyte counts were within the conventional normality limits. Both the absolute quantitative count and its corresponding proportion of neutrophils were within the normal range. The hypersensitive C-reactive protein level was raised to 23.9 mg/L. Comprehensive physical examination, CT scans, and laboratory examination still cannot completely rule out fever caused by bacterial infection. The fever subsided after acetaminophen and anti-infective symptomatic treatment with levofloxacin, but dry mouth and fatigue symptoms persisted. Two days later, the patient developed fever again with a maximum temperature of 38.8 degrees Celsius, and routine blood tests indicated leukocytes at 0.8 × 10^9^/l, neutrophils at 0.03 × 10^9^/l, hemoglobin at 103 g/l, and platelets within the normal range. The patient was given imipenem for preventing infection and granulocyte colony-stimulating factor for leukocyte therapy. On the fifth day of hospitalization, the patient developed a rash (Fig. 1), and a re-examination showed neutrophil counts close to 0/mm3, hemoglobin levels at 89 g/l and IL-6 at 28.4 pg/ml. Tests for COVID-19, EBV DNA, respiratory viruses, CMV, rubella, and herpes viruses all returned negative results.

**Fig. 2:**
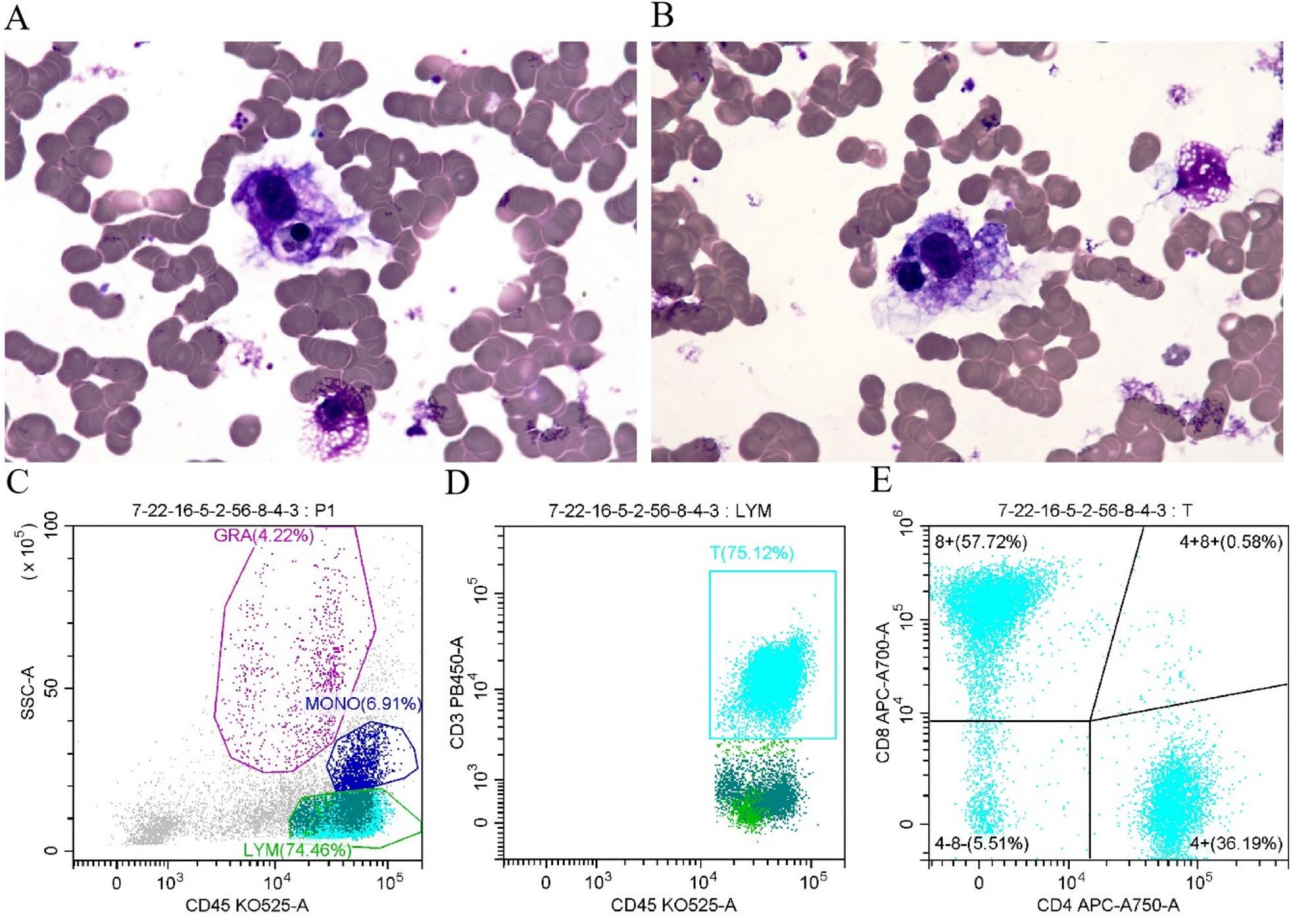
Bone marrow smear diagnosis and flow cytometry detection. **A **Hemophagocytosis in bone marrow and intracytoplasmic orthochromatic normoblast (black arrow); Wright-Giemsa staining, magnification of 400 ×. **B** Hemophagocytosis in bone marrow and intracytoplasmic granulocyte (black arrow); Wright-Giemsa staining, magnification of 400 ×. **C** Ratio of granulocytes, monocytes and lymphocyte in bone marrow detected by flowcytometry. **D** The percentage of T lymphocytes to total lymphocytes in bone marrow detected by flow cytometry. **E **The proportion of CD4 + T lymphocytes and CD8 + T lymphocytes to T lymphocytes

We strongly suspected immune therapy-related granulomatous deficiency with fever and administered intravenous immunoglobulin and methylprednisolone shock therapy, followed by a bone marrow biopsy. The biopsy revealed a number of hemophagocytic cells (Fig. 2), sCD25 (pg/ml) at 7263 (< 6400), NK cell activity (%) at 11.27 (> 15.11), and an ultrasound that did not show splenomegaly, meeting the 6 of the 8 diagnostic criteria in HLH-2004. Upon evaluating both pembrolizumab and bevacizumab using the Naranjo Score (refer to Supplementary data 3), pembrolizumab registered a score of 6, while bevacizumab received a 3. Considering the rarity of HLH as a complication of ICI therapy and the lack of evidence linking anti-vascular therapy to HLH onset—though it may intensify ICI effects—we deduce that pembrolizumab primarily induces HLH, with bevacizumab potentially worsening the syndrome. The patient was diagnosed with hemophagocytic lymphohistiocytosis and treated with methylprednisolone, posaconazole, and caspofungin due to test results indicating a G-test value of 30.70 pg/mL and a GM-test value of Aspergillus galactomannan at 3.230ug/L. After a week of treatment, the patient's body temperature returned to normal.

**Fig. 3:**
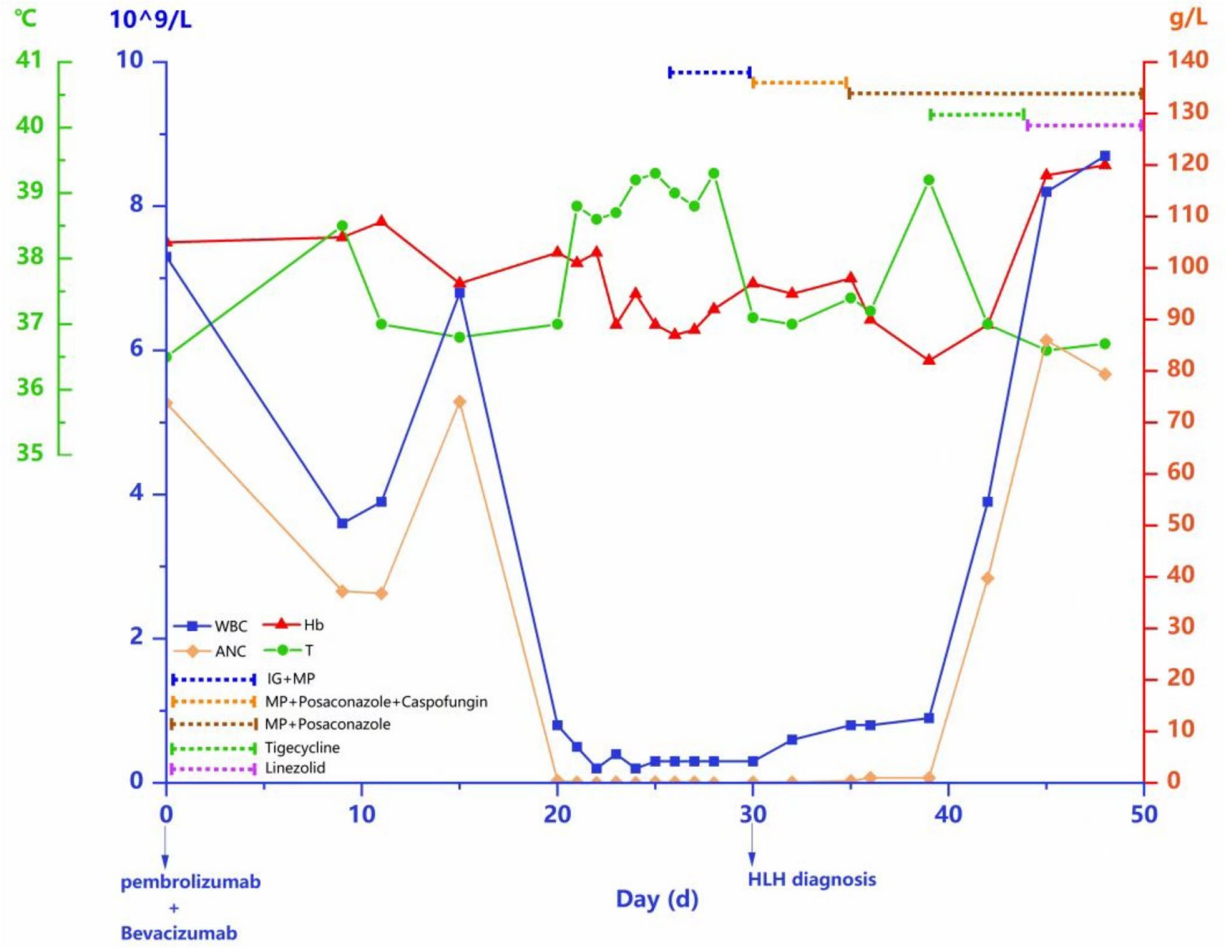
Leukocytes, absolute neutrophil count, hemoglobin and temperature trends during treatment. The X-axis represented the number of days from the patient received pembrolizumab and bevacizumab treatment; The left blue Y-axis responded to the value of leukocytes and absolute neutrophil count; The left green Y-axis responded to the patient's temperature value; The right red Y-axis responded to the patient's hemoglobin value. IG: immunoglobulin MP: methylprednisolone

In the second week, the patient experienced fever again, and a pathogen NGS test suggested Enterococcus faecalis infection, leading to the addition of tigecycline to her treatment. Three days later, the fever disappeared and routine blood tests showed that leukocytes and hemoglobin levels had mostly returned to normal ranges (Fig. 3). The patient was discharged with oral medications methylprednisolone tablets, linezolid tablets, and posaconazole, and fever, rash, hip pain, and bone marrow suppression did not recur. Within six months after the patient's last ICI treatment, the patient did not undergo any antitumor therapy, and the latest follow-up results still showed significant and durable tumor regression without recurrence.

### Systematic review

**Fig. 4:**
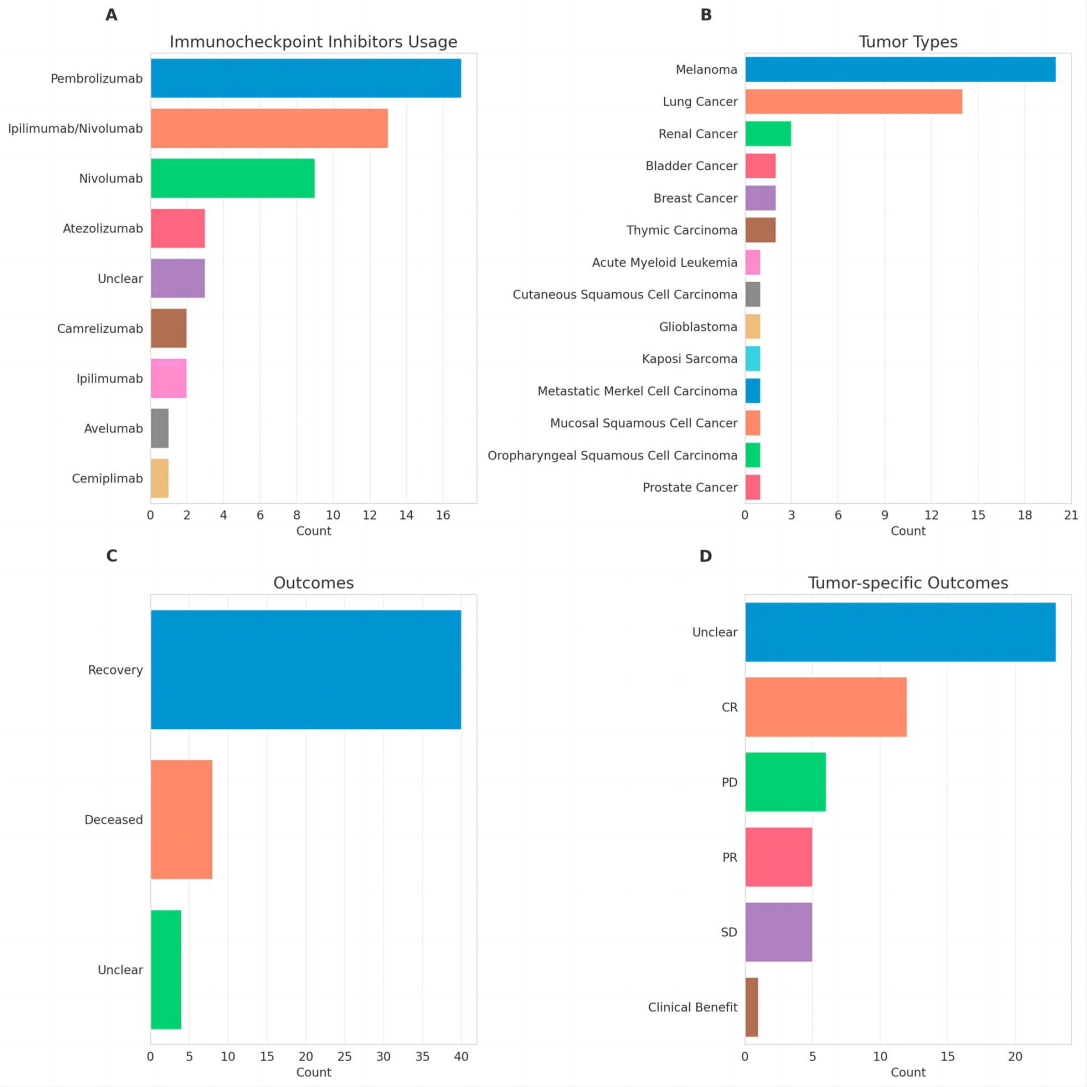
ICI usage and associated clinical data. **A** Counts of various ICIs administered. **B** Number of cases across different tumor types. **C** Patient outcomes post-ICI treatment. **D** Tumor-specific responses following ICI therapy

We analyzed a total of 40 articles, excluding 2 articles from the pharmacovigilance database [3, 9] in the WHO repository for separate discussion due to insufficient clinical information for diagnostic evaluation. From the remaining 38 literature reports, we identified 52 cases of HLH associated with ICIs. The primary characteristics of these cases are summarized in Table 1. Our findings reveal that 28 patients were male and 24 were female, with a median age of onset at 60 years (ranging from 2 to 81 years), indicating that HLH can occur at any age. As noted in the WHO pharmacovigilance database, the age of onset varied considerably, from a young child of 2 years to an elderly individual of 101 years. Immunotherapy was administered for lung cancer (*n* = 13), melanoma (*n* = 20), bladder cancer (*n* = 2), kidney cancer (*n* = 3), and leukemia (*n* = 2) (Fig. 4B), with proportions comparable to those in the pharmacovigilance database [3]. However, no similar cases were reported for cervical cancer.

**Table 1:** Context of HLH

Type and stage	Age and sex	Antibody	Common antitumor treatment	History of autoimmune diseases	ICI cycle	Time- to-symptoms (days)	Year country Ref
Squamous non-small cell lung cancer, stage IV	63 F	Nivolumab	None	None	2	1	2016 Japan [10]
Metastatic melanoma	77 M	Nivolumab	None	Unclear	17 months of therapy	Unclear	2017 France [11]
Metastatic melanoma	42 M	Ipilimumab/nivolumab	None	Unclear	Unclear	Unclear	2017 France [11]
Metastatic Merkel cell carcinoma	81 M	Avelumab	Radiotherapy	Unclear	1	1	2017 France [11]
Bladder, stage IV	76 M	Pembrolizumab	None	None	9 months of therapy	Unclear	2017 USA [12]
Melanoma, stage IV	52 F	Ipilimumab	Radiotherapy	None	1	56	2018 France [13]
Melanoma, stage IV	58 M	Pembrolizumab	None	None	6	31	2018 USA [14]
Melanoma, stage IV	35 F	Ipilimumab/ nivolumab	None	None	1	21	2018 USA [15]
Melanoma, stage IV	26 F	Ipilimumab/ nivolumab	None	Immune thyroiditis	4	7	2018 Germany [16]
Melanoma, stage IV	60 F	Pembrolizumab	Dabrafenib + trametinib	None	Unclear	13	2018 Japan [17]
Thymic carcinoma, stage IV	49 M	Pembrolizumab	None	Psoriasis	1 year of therapy	Unclear	2019 USA [18]
Metastatic breast cancer	58 F	Pembrolizumab	None	None	4	30	2019 USA [19]
Prostate, stage IV	68 M	Pembrolizumab	None	None	Unclear	Unclear	2019 Germany [20]
Lung squamous cell carcinoma, stage IIIB	78 M	Pembrolizumab	None	None	1	10	2019 Japan [21]
Lung pleomorphic adenocarcinoma	52 F	Nivolumab	None	None	4	14	2019 Japan [22]
Melanoma, stage IV	69 F	Nivolumab	None	None	Unclear	30	2019 Australia [23]
Lung adeno- carcinoma, stage IV	78 M	Pembrolizumab	None	None	1	7	2020 Japan [24]
Melanoma, stage IV	42 M	Ipilimumab, nivolumab	None	None	2	Unclear	2020 Switzerland [25]
Melanoma, stage IV	36 M	Nivolumab	None	None	5	Unclear	2020 Switzerland [25]
Melanoma, stage IV	32 M	Ipilimumab, nivolumab	None	None	3	Unclear	2020 Switzerland [25]
Renal cell carcinoma, stage IV	54 M	Nivolumab, ipilimumab	Cabozantinib	No	1	6	2020 UK [26]
Pulmonary sarcomatoid carcinoma, stage IV	54 M	Pembrolizumab	None	Unclear	1	7	2020 France [27]
Melanoma, stage IV	35 F	Ipilimumab, nivolumab	None	Unclear	1	21	2020 France [27]
Melanoma, stage IV	52 F	Ipilimumab, pem brolizumab	None	Unclear	Unclear	30	2020 France [27]
Melanoma, stage IV	69 M	Ipilimumab, nivolumab	None	Unclear	2	Unclear	2020 France [27]
Melanoma, stage IV	27 M	Ipilimumab, nivolumab	None	Unclear	Unclear	Unclear	2020 France [27]
Lung adeno- carcinoma, stage IIIB	74 M	Pembrolizumab	None	Rheumatoid Arthritis	1	27	2020 Japan [28]
Glioblastoma	74 M	Nivolumab	None	None	2	17	2020 USA [29]
Melanoma, stage IV	69 F	Ipilimumab, nivolumab	None	Sarcoidosis	2	1	2020 Japan [30]
Oropharyngeal squamous cell carcinoma, stage IV	61 M	Pembrolizumab	None	None	14	4	2020 USA [31]
Melanoma, stage IV	68 Unclear	Nivolumab	Dabrafenib + trametinib	None	Unclear	21	2020 Germany [32]
Choroidal melanoma, stage IV	75 F	Ipilimumab	None	None	3	Unclear	2021 Spain [33]
Lung adeno- carcinoma, stage IV	75 M	Pembrolizumab	None	Unclear	1	10	2021 Japan [33]
Lung adeno carcinoma. Stage IIIB	60 F	Pembrolizumab	None	Unclear	Unclear	30	2021 Japan [33]
Renal cell carcinoma, stage IV	68 M	Ipilimumab, nivolumab	None	None	Unclear	Unclear	2021 USA [34]
Kaposi sarcoma	85 M	Nivolumab	None	None	9	Unclear	2021 USA [35]
Melanoma, stage IV	57 F	Ipilimumab, nivolumab	None	None	4	Unclear	2021 Poland [36]
Lung adenocarcinoma, stage IV	65F	Atezolizumab	Carboplatin + paclitaxel	Antinuclear antibody/Antidouble-strand DNA antibody positive	Unclear	Unclear	2021 Japan [37]
Lung carcinoma, stage IV	59 F	Unclear	None	None	1	11	2021 UK [38]
Breast cancer, stage IV	42 F	Unclear	None	None	1	11	2021 UK [38]
Bladder cancer, stage IV	67 M	Unclear	None	None	1	10	2021 UK [38]
Melanoma, stage IV	33 M	Ipilimumab, nivolumab	None	None	2	Unclear	2021 USA [39]
Lung adeno- carcinoma, stage IV	36 M	Atezolizumab	Bevacizumab + carboplatin + paclitaxel	None	1	7	2022 Australia [40]
Lung adeno- carcinoma, stage IV	67 M	Atezolizumab	None	Immune thrombocytopenic purpura	1	14	2022 Spain [41]
Renal cell carcinoma, stage III	Unclear F	Nivolumab	Pegilodecakin	None	4	Unclear	2022 USA [42]
Thymic carcinoma, stage IV	50 F	Pembrolizumab	None	Sjögren’s syndrome	1	7	2022 China [43]
Lung squamous carcinoma, stage IV	70 M	Pembrolizumab	None	Antinuclear antibody positive	1	7	2022 China [43]
Cutaneous squamous cell carcinoma, stage IV	80 F	Pembrolizumab	None	None	6	2	2022 USA [44]
Acute Myeloid Leukemia(M4)	10 F	Camrelizumab	Allo-HSCT	Unclear	1	1	2022 China [45]
Acute Myeloid Leukemia(M7)	2 F	Camrelizumab	Allo-HSCT	Unclear	1	1	2022 China [45]
Non-small cell lung carcinoma IV	60 M	Pembrolizumab	Carboplatin, pemetrexed	None	6 months of therapy	6	2023 Japan [46]
Mucosal squamous cell cancer IV	67 F	Cemiplimab	None	None	2	2	2023 USA [47]

*F* female, *M* male, *HLH* hemophagocytic lymphohistiocytosis, *ICI* immune checkpoint inhibitor

The class composition of ICIs primarily included pembrolizumab (*n* = 17), nivolumab (*n* = 9), and nivolumab in combination with ipilimumab (*n* = 13) (Fig. 4A). Other PD-1 or PD-L1 inhibitors, such as cemiplimab and atezolizumab-induced HLH, have been increasingly identified. In the 2018 database, atezolizumab accounted for only 3%, but the latest data show a rise to 12%. Additionally, we found two cases of camrelizumab [45] and one case of avelumab [11], which have not been reported in the databases (Fig. 4A).

Most cases involved single ICI therapy. However, among the combined anti-tumor therapies, two were combined with radiotherapy, two leukemia cases with allogeneic stem cell transplantation, two cases with dabrafenib + trametinib, two cases with anti-angiogenic therapy (cabozantinib and bevacizumab, respectively), and three cases with carboplatin, paclitaxel, and pemetrexed chemotherapy alongside immunotherapy. The number of ICI treatment cycles before the diagnosis of HLH ranged from 1 to 14, but incidences tended to decrease as the number of cycles increased. Excluding cases with missing information, HLH presented in 62% of all cases during the first 1–2 cycles of treatment. The time from the last ICI infusion to HLH onset ranged from 1 to 56 days, with a median of 10 days.

Of the case reports with previous history, 8 (20.5%) patients had previous autoimmune related diseases, such as rheumatoid arthritis, immune thrombocytopenic purpura, Sjögren's syndrome, immune thyroiditis, psoriasis, sarcoidosis, or positive antinuclear antibodies, which is significantly higher than the prevalence of autoimmune diseases in the general population.

The clinical and biological characteristics, as well as the outcomes of the 52 patients, are shown in Table 2. All 52 patients met Hscore > 169 or HLH-2004 criteria. Clinical symptoms included fever in 48 (94.2%) patients, splenomegaly in 26 (50.0%), hepatomegaly in 13 (25%), and skin rash in 9 (19.2%). Excluding cases with missing information, blood tests for most patients revealed pancytopenia or bicytopenia: specifically, pancytopenia in 25 (54.3%) patients, bicytopenia (thrombocytopenia and anemia) in 11 (23.9%), isolated thrombocytopenia in 8 (17.3%), and isolated anemia in 2 (4.3%).

**Table 2:** Clinical characteristics of HLH, treatment and outcome

Clinical features	Biological results	Intervention	outcome	Other immune-related toxicities	Rechallen-ge with ICI	Tumor- Specific outcome	Year country Ref
Fever, rash	Pancytopenia, hyperferritinaemia, hemophagocytosis (BM)	Steroids	Recovery	Stevens–Johnson syndrome, immune-related pneumonitis	No	CR	2016 Japan [10]
Fever	Bicytopenia, hyperferritinaemia, hemophagocytosis (BM)	Steroids, Abs	Deceased	None	No	Unclear	2017 France [11]
Fever, hepatomegaly, rash	Bicytopenia, hyperferritinaemia hemophagocytosis (BM)	Steroids, Abs	Recovery	None	No	PD	2017 France [11]
Fever, hepatomegaly	Bicytopenia, hyperferritinaemia hemophagocytosis (BM)	Steroids	Deceased	None	No	Unclear	2017 France [11]
Fever, tachycardia, rash, splenomegaly	Pancytopenia, hyperferritinaemia, hypofibrinogenemia, acute renal failure, ↗sCD25, ↘NK cell function, hemophagocytosis (BM)	Steroids, etoposide	Unclear	None	Unclear	CR	2017 USA [12]
Fever	Pancytopenia, hyperferritinaemia, hemophagocytosis (BM)	Steroids, etoposide	Deceased	Immune-related hepatitis	No	Unclear	2018 France [13]
Fever	Bicytopenia (thrombopenia and anemia), hyperferritinaemia, ↗sCD163, ↘NK cell function, hemophagocytosis (BM)	Steroids	Recovery	None	No	CR for 1 year	2018 USA [14]
Tachycardia, hypotension, splenomegaly	Bicytopenia (thrombopenia and anemia), hyperferritinaemia, ↗sCD25, hemophagocytosis (BM)	Steroids	Recovery	None	No	CR	2018 USA [15]
Fever	Pancytopenia, hyperferritinaemia, hypofibrinogenemia, ↗sCD25, hemophagocytosis (BM)	Steroids	Recovery	Immune-related hepatitis	No	CR	2018 Germany [16]
Fever, hepatosplenomegaly, erythema multiforme-like	Pancytopenia, hyperferritinaemia, hemophagocytosis (BM)	Steroids	Recovery	None	No	PR	2018 Japan [17]
Fever, neurological involvement	Pancytopenia, hyperferritinaemia, ↗sCD25, hemophagocytosis (BM)	unclear	Deceased	Immune-related encephalitis	No	Clinical benefit	2019 USA [18]
Fever, rash	Bicytopenia (thrombopenia and anemia), hyperferritinaemia, hypofibrinogenemia, ↗sCD25, hemophagocytosis (BM)	Steroids	Recovery	None	No	CR	2019 USA [19]
Fever, hepatosplenomegaly	Pancytopenia, hyperferritinaemia, hypofibrinogenemia, ↗sCD25, ↘NK cell function, hemophagocytosis (BM)	Steroids, plasmapheresis, tacrolimus	Recovery	None	No	CR	2019 Germany [20]
Fever, splenomegaly	Anemia, hyperferritinaemia, ↗sCD25, hemophagocytosis (BM)	Steroids, Abs	Recovery	Autoimmune hemolytic anemia	No	CR	2019 Japan [21]
Fever, purpura fulminans	Thrombocytopenia, hyperferritinaemia, ↗sCD25, acute renal failure no BM examination	Steroids, thrombomodulin, mycophenolate mofetil	Recovery	Immune-related myocarditis	No	CR	2019 Japan [22]
Fevers, hepatosplenomegaly	Bicytopenia (thrombopenia and anemia), hyperferritinaemia, hypofibrinogenemia, ↗sCD25, ↘NK cell function, hemophagocytosis (BM)	Steroids	Recovery	None	No	SD	2019 Australia [23]
Fever	Thrombocytopenia, hypofibrinogenemia, hyperferritinaemia, ↗sCD25, hemophagocytosis (BM)	Steroids, Abs	Recovery	Immune-related hepatitis	No	SD for 3 months	2020 Japan [24]
Fever	Pancytopenia, hyperferritinaemia, hypofibrinogenemia, hemophagocytosis (BM)	Steroids, tocilizumab, plasmapheresis	Recovery	None	No	Unclear	2020 Switzerland [25]
Fever, splenomegaly	Pancytopenia, hyperferritinaemia, hypofibrinogenemia. no BM examination	Steroids, tocilizumab, low dose heparin prophylaxis	Recovery	None	No	Unclear	2020 Switzerland [25]
Fever, hepatosplenomegaly	Pancytopenia, hyperferritinaemia, hypofibrinogenemia, hemophagocytosis (BM)	Steroids, tocilizumab, plasmapheresis, low dose heparin prophylaxis	Recovery	None	No	Unclear	2020 Switzerland [25]
Fever, rash	Bicytopenia (thrombopenia and anemia), hyperferritinaemia, hemophagocytosis (BM)	Steroids, Abs, anakinra	Recovery	None	No	Unclear	2020 UK [26]
Fever, asthenia, dyspnea	Bicytopenia (thrombopenia and anemia), hyperferritinaemia, no BM examination	Steroids, broad-spectrum Abs	Recovery	None	No	PD	2020 France [27]
Asthenia, splenomegaly	Pancytopenia, hyperferritinaemia, hypofibrinogenemia, hemophagocytosis (BM)	Steroids, etoposide, intravenous immune- globulins, tocilizumab	Recovery	None	Yes	SD	2020 France [27]
Fever, asthenia	Pancytopenia, hypofibrinogenemia, hyperferritinaemia, hemophagocytosis (BM)	Steroids, etoposide	Deceased	Immune-related hepatitis	No	PD	2020 France [27]
Fever, splenomegaly	Bicytopenia (thrombopenia and anemia), hyperferritinaemia, no hemophagocytosis on BM smear	Steroids	Recovery	Hepatic cytolysis and lymphocytic meningitis	Yes	PD	2020 France [27]
Fever, splenomegaly	Anemia, hyperferritinaemia, hemophagocytosis (BM)	MTP	Recovery	Hypophysitis, lymphocytic meningitis, colitis, hepatic cytolysis	No	Unclear	2020 France [27]
Fever, joint swelling, rash, hepatosplenomegaly	Pancytopenia, hyperferritinaemia, hemophagocytosis (BM)	Steroids, recombinant thrombomodulin, G-CSF, Abs, etoposide	Recovery	None	No	CR	2020 Japan [28]
Fever, altered mental status, neurological involvement, hepatosplenomegaly	Bicytopenia (thrombopenia and anemia), hyperferritinaemia, ↗sCD25, hemophagocytosis (BM)	Steroids, Abs	Deceased	Immune-related hepatitis, immune-related encephalitis	No	Unclear	2020 USA [29]
Fever, hepatosplenomegaly	Thrombopenia, liver dysfunction, hemophagocytosis (BM)	Steroids, NSAID, Abs	Unclear	Immune-related hepatitis	No	Unclear	2020 Japan [30]
Fever, hepatomegaly	Pancytopenia, hyperferritinaemia, hypofibrinogenemia, ↗sCD25, ↘NK cell function, hemophagocytosis (BM)	Steroids, Abs, etoposide	Recovery	Immune-related hepatitis	Yes	SD	2020 USA [31]
Fever, splenomegaly	Pancytopenia, hyperferritinaemia, hypofibrinogenemia, ↗sCD25, no hemophagocytosis on BM smear	Steroids,broad-spectrum Abs	Recovery	None	No	PR	2020 Germany [32]
Fever, splenomegaly	Bicytopenia (thrombopenia and anemia), hyperferritinaemia, hypofibrinogenemia, hemophagocytosis (BM)	Steroids, tocilizumab	Recovery	None	No	PR	2021 Spain [33]
Fever	Cytopenia(unclear), hyperferritinaemia, hemophagocytosis (BM)	Steroids	Unclear	None	No	Unclear	2021 Japan [33]
None	Cytopenia(unclear), hyperferritinaemia, no BM examination	Steroids	Unclear	None	No	Unclear	2021 Japan [33]
Fever, splenomegaly	Pancytopenia, hyperferritinaemia, hypofibrinogenemia, ↗sCD25, ↘NK cell function, hemophagocytosis (BM)	Steroids	Recovery	None	No	Unclear	2021 USA ( [48]
Fever, rash	Pancytopenia, hyperferritinaemia, hemophagocytosis (BM)	Steroids	Deceased	Immune-related hepatitis	No	Unclear	2021 USA [35]
Fever, general malaise, dyspnea, splenomegaly	Bicytopenia (thrombopenia and anemia), hyperferritinaemia, hypofibrinogenemia, no BM examination	Steroids, FFP, mycophenolate mofetil, cyclophos- phamide, etoposide, ciclosporin	Recovery	None	No	PR	2021 Poland [36]
Fever, splenomegaly	Pancytopenia, hyperferritinaemia, hypofibrinogenaemia, ↗sCD25, ↘NK cell function, hemophagocytosis (BM)	Steroids	Recovery	Autoimmune hemolytic anemia	No	CR	2021 Japan [37]
Fever	Thrombocytopenia, hyperferritinaemia, hemophagocytosis (BM)	Steroids, Abs	Recovery	None	No	Unclear	2021 UK [38]
Fever, maculopapular rash, dyspnea, hypoxia	Thrombocytopenia, hyperferritinaemia, no BM examination	Abs, steroids, tocilizumab	Recovery	None	No	Unclear	2021 UK [38]
Fever	Thrombocytopenia, hyperferritinaemia, hemophagocytosis (BM)	Abs, steroids, tocilizumab, siltuximab, anakinra, plasma exchange, intravenous immunoglobulins	Recovery	None	No	Unclear	2021 UK [38]
Fever, hepatosplenomegaly, neurological involvement	Thrombocytopenia, hyperferritinaemia, hypofibrinogenemia ↗sCD25, hemophagocytosis (BM)	Steroids, Abs	Recovery	Immune-related hepatitis, immune-related encephalitis	No	Unclear	2021 USA [39]
Fever, erythematous, neurological involvement	Thrombocytopenia, hyperferritinaemia, hemophagocytosis (BM)	Steroids, tocilizumab, immunoglobulin	Recovery	None	No	PD	2022 Australia [49]
Fever, asthenia, myalgia, hepatosplenomegaly, neurological involvement	Pancytopenia, acute renal failure, hyperferritinaemia, hypofibrinogenemia, ↗sCD25, hemophagocytosis (BM)	Steroids, tocilizumab, etoposide	Deceased	Immune-related encephalitis	No	PD	2022 Spain [41]
Fever, splenomegaly	Pancytopenia, hyperferritinaemia, ↗sCD25, hemophagocytosis (BM)	Steroids, etoposide	Recovery	Autoimmune hemolytic anemia	No	SD for 1 year	2022 USA [42]
Fever, splenomegaly	Thrombocytopenia, hyperferritinaemia, hypofibrinogenemia, ↗sCD25, ↘NK cell function, no BM examination	Steroids, etoposide	Recovery	None	No	CR	2022 China [43]
Fever, splenomegaly	Abnormal liver function, hyperferritinaemia, ↗sCD25, hemophagocytosis (BM)	Steroids, etoposide	Recovery	None	No	Unclear	2022 China [43]
Fever, splenomegaly	Pancytopenia, hyperferritinaemia, hypofibrinogenemia, ↗sCD25, ↘NK cell function, hemophagocytosis (BM)	Steroids, tocilizumab, etoposide	Recovery	Immune-related hepatitis	No	PR	2022 USA [44]
Fever, rash, diarrhea,jaundice	Pancytopenia, hyperferritinaemia, hypofibrinogenemia, ↗sCD25, ↘NK cell function, no hemophagocytosis on BM smear	Methotrexate, MP,basiliximab	Recovery	Acute graft versus host disease	No	Unclear	2022 China [45]
Fever, rash	Pancytopenia, hyperferritinaemia, ↗sCD25, ↘NK cell function, no hemophagocytosis on BM smear	Methotrexate, MP,basiliximab	Recovery	Acute graft versus host disease	No	Unclear	2022 China [45]
Fever, hepatosplenomegaly	Pancytopenia, hyperferritinaemia, ↗sCD25, hemophagocytosis (BM)	Steroids	Recovery	None	No	Unclear	2023 Japan [46]
Fever	Pancytopenia, hyperferritinaemia, hypofibrinogenemia, ↗sCD25, hemophagocytosis (BM)	Steroids, plasma exchange	Recovery	None	No	Unclear	2023 USA [50]

*BM* bone marrow, *CR* complete response, *PD* progressive disease, *PR* partial response, *SD* stable disease, *Abs* antibiotics, *MTP* methylprednisolone

20 (38.4%) patients experienced other immune-related toxicities, including immune-related hepatitis (*n* = 12), autoimmune hemolytic anemia (*n* = 3), immune-related encephalitis (*n* = 4), immune-related myocarditis (*n* = 1), immune-related pneumonitis (*n* = 1), Stevens-Johnson syndrome (*n* = 1), and two cases of AML after Allo-HSCT presenting with acute graft-versus-host disease. In terms of treatment, nearly all cases received various types of corticosteroids, 13 (25%) patients were treated with etoposide in combination, 10 (19.2%) patients received combined tocilizumab treatment, and other treatments included intravenous immunoglobulins, plasmapheresis, mycophenolate mofetil, and tacrolimus, following some of the protocols in the HLH-2004 treatment criteria [7].

Of the 52 patients, 40 (76.9%) made a full recovery after treatment, while 8 (15.3%) eventually died (Fig. 4C). ICI rechallenge was utilized in 3 cases without any associated adverse outcomes. Despite experiencing severe immune-related adverse effects, most patients had favorable tumor-specific outcomes after immunotherapy. In the 29 cases with tumor outcome information, 12 (41.4%) achieved complete response (CR), 5 (17.2%) partial response (PR), 5 (17.2%) stable disease (SD), and only 6 (20.1%) progressive disease (PD) (Fig. 4D).

Upon follow-up, we observed an interesting phenomenon consistent with our case: 8 cases reported that ICI-induced HLH produced significant and long-lasting responses to the tumor. After the last ICI treatment, the tumor continued to shrink or remained stable on multiple reviews, even without any other anti-tumor therapies.

## Discussion

Hemophagocytic lymphohistiocytosis (HLH) is a severe, hyperinflammatory syndrome triggered by aberrantly activated macrophages and cytotoxic T lymphocytes (CTLs) [51]. In adults, it frequently arises in contexts like untreated hematologic malignancies, chronic rheumatic diseases, or conditions of immunosuppression. The pathogenesis of HLH involves excessive activation of CTLs and their consequential depletion of IL-2, potentially leading to regulatory T cell (Treg) dysfunction [52, 53]. This cascade is pivotal in HLH development. Integral to ICI-based cancer immunotherapy is the simultaneous inhibition of Tregs and activation of CTLs [54]. ICIs enhance T-cell activation and proliferation while impairing Treg cell functionality. By inhibiting immune checkpoint molecules, ICIs prevent tumor cells from evading immune detection [54]. This same mechanism, however, disrupts peripheral T-cell tolerance, fostering the rapid diversification and clonal expansion of potentially toxic cells, which can culminate in hyperinflammation and autoimmunity [55, 56]. Furthermore, in HLH, CTL activation is perpetuated by a macrophage/monocyte expansion loop [51]. Studies in mouse and human cancers indicate that tumor-associated macrophage PD-1 expression inversely correlates with its phagocytic efficacy against tumor cells. Accordingly, PD-1/PD-L1 blockade has been observed to enhance macrophage-mediated phagocytosis of tumor cells [57]. These studies offer insights into the specific mechanisms of HLH associated with immunotherapy and confirm that immunotherapy-related HLH is a genuine and serious complication.

ICI becomes increasingly popular in treating various cancers, the number of immune-related adverse event (irAE) reports has also grown exponentially [58]. For instance, there were only 38 cases of ICI-associated HLH in VigiBase before 2019, but the latest study showed 177 cases in VigiBase. Our brief review suggests that ICI-associated HLH can occur across a broader range of patient populations and cancer types, including the first-ever reported case in cervical cancer, which we found outside the database. The latest VigiBase data on age at onset aligns with the findings from our literature collection, indicating a median age of onset around 60 years. Furthermore, very young onset cases were also found in both our systematic review and the database, with patients as young as two years old. In our analysis, melanoma and lung cancer have emerged as the most common cancer types associated with ICI-related HLH. The predominance of these cancers in HLH cases can be linked to the early approval of ICIs for their treatment, leading to a more extensive pool of data and reported cases. Therapies like pembrolizumab, nivolumab, and their combination with ipilimumab, predominantly used in melanoma and lung cancer, have been well-documented in this regard. However, as the spectrum of approved ICIs expands, an increase in HLH incidence is being observed in other cancers. This trend reflects the evolving landscape of ICI usage and its associated irAEs. Notable among these are various types of acute myeloid leukemia (AML) [45], as well as previously undetected cases in squamous cell skin cancer [44], Kaposi sarcoma [35] and other cancer types. While melanoma and lung cancer currently have a higher number of HLH cases due to their early inclusion in ICI therapy, the rising number of HLH cases in cancers like AML, squamous cell skin cancer, and Kaposi sarcoma, as seen with newer ICIs like camrelizumab and avelumab, indicates a broader oncological concern. Identifying specific cancer types or ICI agents that are more prone to inducing HLH remains a challenge, necessitating further research to clarify these associations and improve our understanding and management of ICI-related HLH across different cancer scenarios.

Our case involved the combination of bevacizumab for anti-angiogenic therapy, and prior to this, there were two reported cases of HLH resulting from ICI combined with anti-angiogenic therapy. One of these previous cases suggested that the development of HLH was closely linked to anti-angiogenic therapy [26]. Several studies using the FDA adverse event reporting system (FAERS) database have demonstrated that bevacizumab combined with PD-1 monoclonal antibody increases the risk of serious adverse effects such as fever, physical condition deterioration, thrombocytopenia, bone marrow failure, and neutropenia in oncology patients [59, 60]. Regarding specific mechanisms, VEGF can inhibit T cell function, increase Tregs and MDSCs, and hinder the differentiation and activation of DCs [61]. However, the number of HLH cases due to ICI combined with anti-vascular therapy remains low, and further confirmation of the relevance is needed.

In our case, the patient had a history of psoriasis, and our case review found that 20.5% of patients with ICI-related HLH had a history of autoimmune disease. Recent studies have demonstrated that pre-existing autoantibodies (including antithyroid [62], antinuclear [63] and other autoimmune-related antibodies [64]) are strong biomarkers for irAE, and autoimmune diseases such as rheumatoid arthritis and psoriasis have been shown to confer an elevated risk of irAEs [65]. We suggest that, in HLH as in other irAEs, patients with autoantibodies and autoimmune diseases are likely to be at a higher risk of developing the disease.

Among the symptoms exhibited by the patient, fever remains the most common. Interestingly, in terms of laboratory tests, there is a difference from our case compared to most of the reported cases, as they often experienced a drop in platelets as the first symptom. However, our case showed granulocyte deficiency as the initial abnormal laboratory test, and the platelet count remained normal from the onset of HLH to the end. No laboratory test results similar to ours have been found in the cases collected so far.

Regarding the treatment of HLH, patients (*n* = 25 or 48%) used steroids alone or in combination with antibiotics. Typically, patients with HLH refractory to steroids are treated with immunosuppressive drugs such as cyclophosphamide, or with etoposide, a drug that may even be used as a front-line treatment in immunosuppressed patients [66]. IL-6 inhibition with specific anti-IL-6 receptor antibodies, such as tocilizumab, has proven highly effective against cytokine release syndrome [67]. One study suggested that IL-6 blockade administered alongside ICIs can ameliorate irAEs while enhancing the antitumoral effect of ICIs [68]. In the 10 cases we collected where tocilizumab was used, only 1 death occurred, which may indicate the effectiveness of tocilizumab in treating HLH. However, confirmation through larger-scale case information is needed.

The prognosis of ICI-associated HLH is relatively favorable, with only 15.3% mortality in the pooled cases, which is much better than the 41% mortality reported in other types of HLH [2]. However, among the four patients who developed immune-associated encephalitis, we found three deaths, suggesting that immune-associated encephalitis is a significant factor contributing to a poor prognosis.

ICI-associated HLH is undoubtedly a challenging complication that clinicians strive to avoid. However, we observed that patients who recover from it may experience improved tumor control. In recent years, numerous studies have demonstrated that irAEs may lead to a better prognosis for tumor treatment [58], but the link between high-grade irAEs and tumor prognosis remains poorly understood. A retrospective study based on a small sample showed that patients with grade 3 or higher irAEs had longer overall survival (OS) than those with grade 1 or 2 irAEs [69]. Additionally, the results of a recent study, which combined data from three large clinical trials, revealed that grade 3–4 irAEs had a favorable prognostic effect over time compared to patients without irAEs [70]. According to our summary data, ICI-associated HLH tended to have a very positive effect on tumor outcome, with varying degrees of tumor regression and stabilization in 80% of cases with available prognosis information. This effect was reported to persist after a period of follow-up in 8 cases, which aligns with our own case suggesting that recovery from ICI-associated HLH can have a significant impact on tumor tissue. This phenomenon was first proposed in 2016 by M Takeshita et al. [10], who discovered the coincidence of immunotherapy-associated hemophagocytic syndrome and rapid tumor regression. However, the exact mechanism still requires further investigation, as the number of cases is small. Nonetheless, the exact mechanism behind this observation warrants further investigation, given the limited number of cases currently available for study. It is crucial to continue researching this topic, taking into account that the primary goal of medical professionals is to avoid complications like ICI-associated HLH and provide the most effective and safe treatment for patients.

In conclusion, ICI-associated HLH may occur in any ICI and in any cancer, and combined anti-vascular therapy and the presence of a history of autoimmune disease may increase the incidence of HLH, with complete recovery in most cases of HLH after steroid-based therapy, and may have a favourable impact on tumour outcome.

## Conclusions

HLH, a relatively understudied complication, tends to occur more frequently in patients with a history of autoimmune diseases. Our findings indicate that recovery from HLH during treatment may correlate with improved survival outcomes, underlining the need for further research in this area.

### Supplementary Information

**Additional file 1.**

**Additional file 2.**

**Additional file 3.**

## Data Availability

All data generated or analysed during this study are included in this published article [and its supplementary information files].

## References

[1] Postow MA, Sidlow R, Hellmann MD (2018). Immune-related adverse events associated with immune checkpoint blockade. N Engl J Med.

[2] Ramos-Casals M, Brito-Zerón P, López-Guillermo A, Khamashta MA, Bosch X (2014). Adult haemophagocytic syndrome. Lancet.

[3] Diaz L, Jauzelon B, Dillies AC, Le Souder C, Faillie JL, Maria ATJ, Palassin P. Hemophagocytic Lymphohistiocytosis Associated with Immunological Checkpoint Inhibitors: A Pharmacovigilance Study. J Clin Med. 2023;12. 10.3390/jcm12051985. 10.3390/jcm12051985 PMC10004618 36902771

[4] Brahmer JR, Abu-Sbeih H, Ascierto PA, Brufsky J, Cappelli LC, Cortazar FB, Gerber DE, Hamad L, Hansen E, Johnson DB, et al. Society for Immunotherapy of Cancer (SITC) clinical practice guideline on immune checkpoint inhibitor-related adverse events. J Immunother Cancer. 2021;9. 10.1136/jitc-2021-002435. 10.1136/jitc-2021-002435 PMC8237720 34172516

[5] Naumann RW, Hollebecque A, Meyer T, Devlin MJ, Oaknin A, Kerger J, López-Picazo JM, Machiels JP, Delord JP, Evans TRJ (2019). Safety and Efficacy of Nivolumab Monotherapy in Recurrent or Metastatic Cervical, Vaginal, or Vulvar Carcinoma: Results From the Phase I/II CheckMate 358 Trial. J Clin Oncol.

[6] Chung HC, Ros W, Delord JP, Perets R, Italiano A, Shapira-Frommer R, Manzuk L, Piha-Paul SA, Xu L, Zeigenfuss S (2019). Efficacy and Safety of Pembrolizumab in Previously Treated Advanced Cervical Cancer: Results From the Phase II KEYNOTE-158 Study. J Clin Oncol.

[7] Henter JI, Horne A, Aricó M, Egeler RM, Filipovich AH, Imashuku S, Ladisch S, McClain K, Webb D, Winiarski J (2007). HLH-2004: Diagnostic and therapeutic guidelines for hemophagocytic lymphohistiocytosis. Pediatr Blood Cancer.

[8] Fardet L, Galicier L, Lambotte O, Marzac C, Aumont C, Chahwan D, Coppo P, Hejblum G (2014). Development and validation of the HScore, a score for the diagnosis of reactive hemophagocytic syndrome. Arthritis Rheumatol.

[9] Noseda R, Bertoli R, Müller L, Ceschi A (2019). Haemophagocytic lymphohistiocytosis in patients treated with immune checkpoint inhibitors: analysis of WHO global database of individual case safety reports. J Immunother Cancer.

[10] Takeshita M, Anai S, Mishima S, Inoue K (2017). Coincidence of immunotherapy-associated hemophagocytic syndrome and rapid tumor regression. Ann Oncol.

[11] Malissen N, Lacotte J, Du-Thanh A, Gaudy-Marqueste C, Guillot B, Grob JJ (2017). Macrophage activation syndrome: a new complication of checkpoint inhibitors. Eur J Cancer.

[12] Shah D, Shrestha R, Ramlal R, Hatton J, Saeed H (2017). Pembrolizumab associated hemophagocytic lymphohistiocytosis. Ann Oncol.

[13] Michot JM, Pruvost R, Mateus C, Champiat S, Voisin AL, Marabelle A, Lambotte O (2018). Fever reaction and haemophagocytic syndrome induced by immune checkpoint inhibitors. Ann Oncol.

[14] Sadaat M, Jang S (2018). Hemophagocytic lymphohistiocytosis with immunotherapy: brief review and case report. J Immunother Cancer.

[15] Hantel A, Gabster B, Cheng JX, Golomb H, Gajewski TF (2018). Severe hemophagocytic lymphohistiocytosis in a melanoma patient treated with ipilimumab + nivolumab. J Immunother Cancer.

[16] Satzger I, Ivanyi P, Länger F, Kreipe HH, Schaper-Gerhardt K, Beutel G, Cornberg M, Gutzmer R (2018). Treatment-related hemophagocytic lymphohistiocytosis secondary to checkpoint inhibition with nivolumab plus ipilimumab. Eur J Cancer.

[17] Sasaki K, Uehara J, Iinuma S, Doi H, Honma M, Toki Y, Ishida-Yamamoto A (2018). Hemophagocytic lymphohistiocytosis associated with dabrafenib and trametinib combination therapy following pembrolizumab administration for advanced melanoma. Ann Oncol.

[18] Laderian B, Koehn K, Holman C, Lyckholm L, Furqan M (2019). Association of Hemophagocytic Lymphohistiocytosis and Programmed Death 1 Checkpoint Inhibitors. J Thorac Oncol.

[19] Al-Samkari H, Snyder GD, Nikiforow S, Tolaney SM, Freedman RA, Losman JA (2019). Haemophagocytic lymphohistiocytosis complicating pembrolizumab treatment for metastatic breast cancer in a patient with the PRF1A91V gene polymorphism. J Med Genet.

[20] Lorenz G, Schul L, Bachmann Q, Angermann S, Slotta-Huspenina J, Heemann U, Küchle C, Schmaderer C, Jäger M, Tauber R (2019). Hemophagocytic lymphohistiocytosis secondary to pembrolizumab treatment with insufficient response to high-dose steroids. Rheumatology (Oxford).

[21] Okawa S, Kayatani H, Fujiwara K, Ozeki T, Takada K, Iwamoto Y, Minami D, Sato K, Shibayama T (2019). Pembrolizumab-induced Autoimmune Hemolytic Anemia and Hemophagocytic Lymphohistiocytosis in Non-small Cell Lung Cancer. Intern Med.

[22] Honjo O, Kubo T, Sugaya F, Nishizaka T, Kato K, Hirohashi Y, Takahashi H, Torigoe T (2019). Severe cytokine release syndrome resulting in purpura fulminans despite successful response to nivolumab therapy in a patient with pleomorphic carcinoma of the lung: a case report. J Immunother Cancer.

[23] Chin CK, Hall S, Green C, Van Hazel G, Spagnolo D, Cheah CY (2019). Secondary haemophagocytic lymphohistiocytosis due to checkpoint inhibitor therapy. Eur J Cancer.

[24] Takahashi H, Koiwa T, Fujita A, Suzuki T, Tagashira A, Iwasaki Y (2020). A case of pembrolizumab-induced hemophagocytic lymphohistiocytosis successfully treated with pulse glucocorticoid therapy. Respir Med Case Rep.

[25] Özdemir BC, Latifyan S, Perreau M, Fenwick C, Alberio L, Waeber G, Spertini F, de Leval L, Michielin O, Obeid M (2020). Cytokine-directed therapy with tocilizumab for immune checkpoint inhibitor-related hemophagocytic lymphohistiocytosis. Ann Oncol.

[26] Azari AE, Stratton R, Singh A (2021). First case of hemophagocytic lymphohistiocytosis secondary to cabozantinib with checkpoint inhibitors. Rheumatology (Oxford).

[27] Dupré A, Michot JM, Schoeffler A, Frumholtz L, Baroudjian B, Delyon J, Lebbe C, Lambotte O (2020). Haemophagocytic lymphohistiocytosis associated with immune checkpoint inhibitors: a descriptive case study and literature review. Br J Haematol.

[28] Akagi Y, Awano N, Inomata M, Kuse N, Tone M, Yoshimura H, Jo T, Takada K, Kumasaka T, Izumo T (2020). Hemophagocytic Lymphohistiocytosis in a Patient with Rheumatoid Arthritis on Pembrolizumab for Lung Adenocarcinoma. Intern Med.

[29] Thummalapalli R, Heumann T, Stein J, Khan S, Priemer DS, Duffield AS, Laterra J, Couzi R, Lim M, Holdhoff M (2020). Hemophagocytic Lymphohistiocytosis Secondary to PD-1 and IDO Inhibition in a Patient with Refractory Glioblastoma. Case Rep Oncol.

[30] Mizuta H, Nakano E, Takahashi A, Koyama T, Namikawa K, Yamazaki N (2020). Hemophagocytic lymphohistiocytosis with advanced malignant melanoma accompanied by ipilimumab and nivolumab: A case report and literature review. Dermatol Ther.

[31] Kalmuk J, Puchalla J, Feng G, Giri A, Kaczmar J (2020). Pembrolizumab-induced Hemophagocytic Lymphohistiocytosis: an immunotherapeutic challenge. Cancers Head Neck.

[32] Dudda M, Mann C, Heinz J, Schmidgen I, Weid F, Kühn M, Saloga J, Grabbe S, Loquai C (2021). Hemophagocytic lymphohistiocytosis of a melanoma patient under BRAF/MEK-inhibitor therapy following anti-PD1 inhibitor treatment: a case report and review to the literature. Melanoma Res.

[33] Olivares-Hernández A, Figuero-Pérez L, Amores Martín MA, Bellido Hernández L, Mezquita L, Vidal Tocino MDR, López Cadenas F, Gómez-Caminero López F, Escala-Cornejo RA, Cruz Hernández JJ (2021). Response to Treatment with an Anti-Interleukin-6 Receptor Antibody (Tocilizumab) in a Patient with Hemophagocytic Syndrome Secondary to Immune Checkpoint Inhibitors. Case Rep Oncol Med.

[34] Masood A, Wahab A, Clifford T, Weaver EJ, Ehsan H, El Ayass W (2021). Secondary hemophagocytic lymphohistiocytosis due to nivolumab/ipilimumab in a renal cell cancer patient-A case report. Clin Case Rep.

[35] Choi S, Zhou M, Bahrani E, Martin BA, Ganjoo KN, Zaba LC (2021). Rare and fatal complication of immune checkpoint inhibition: a case report of haemophagocytic lymphohistiocytosis with severe lichenoid dermatitis. Br J Haematol.

[36] Pacholczak-Madej R, Grela-Wojewoda A, Lompart J, Żuchowska-Vogelgesang B, Ziobro M (2022). Effective Treatment of a Melanoma Patient with Hemophagocytic Lymphohistiocytosis after Nivolumab and Ipilimumab Combined Immunotherapy. Prague Med Rep.

[37] Endo Y, Inoue Y, Karayama M, Nagata Y, Hozumi H, Suzuki Y, Furuhashi K, Enomoto N, Fujisawa T, Nakamura Y (2022). Marked, Lasting Disease Regression and Concomitantly Induced Autoimmune Hemolytic Anemia and Hemophagocytic Lymphohistiocytosis in a Patient With Lung Adenocarcinoma and Autoantibodies Receiving Atezolizumab Plus Chemotherapy: A Case Report. JTO Clin Res Rep.

[38] Tiu C, Shinde R, Pal A, Biondo A, Lee A, Tunariu N, Jhanji S, Grover V, Tatham K, Gruber P (2021). A wolf in sheep's clothing: systemic immune activation post immunotherapy. J Immunother Precis Oncol.

[39] Ghous G, Shoukat HMH, Tarar ZI, Zafar MU, McGreevy JW (2021). Encephalitis Associated With Hemophagocytic Lymphohistiocytosis Secondary to Immune Checkpoint Inhibitors: An Unfamiliar Spin-Off. Cureus.

[40] Heynemann S, Vanguru V, Adelstein S, et al. Hemophagocytic lymphohistiocytosis (HLH) and cytokine release syndrome (CRS) in a patient with oncogene-addicted metastatic non-small cell lung cancer (NSCLC) following combination chemotherapy-immunotherapy. Asia Pac J Clin Oncol. 2022. 10.1111/ajco.13906 36562695

[41] Rubio-Perez J, Rodríguez-Perez ÁR, Díaz-Blázquez M, Moreno-García V, Dómine-Gómez M (2022). Treatment-related hemophagocytic lymphohistiocytosis due to atezolizumab: a case report and review of the literature. J Med Case Rep.

[42] Nelson BE, Ejezie CL, Stephen BA, Nardo M, Campbell E, Gong J, Hong DS, Fu S, Yap TA, Murphy MB (2022). Spectrum of immune checkpoint inhibitor anemias: results from a single center, early-phase clinical trials case series experience. J Hematol.

[43] Wei Y, He W, Sun W, Wu C, Ren D, Wang X, Zhang M, Huang M, Ji N (2022). Hemophagocytic lymphohistiocytosis in two patients following treatment with pembrolizumab: two case reports and a literature review. Transl Cancer Res.

[44] Marar R, Prathivadhi-Bhayankaram S, Krishnan M (2022). Immune Checkpoint Inhibitor-Induced Hemophagocytic Lymphohistiocytosis in a Patient With Squamous Cell Carcinoma. J Hematol.

[45] Du ZZ, Zhou M, Ling J, Cao L, Kong L, Cheng S, Xiao P, Hu S (2022). PD-1 Checkpoint Blockade in Patients for Acute Myeloid Leukemia after HSCT Relapse Resulted in Severe GVHD and sHLH. Case Rep Hematol.

[46] Oyama S, Shirai T, Abe Y, Tsuchiya M, Inui T, Suhara K, Noto S, Kamimura M (2023). Immune checkpoint inhibitor-related haemophagocytic lymphohistiocytosis in a patient with non-small cell lung carcinoma. Respirol Case Rep.

[47] Martin-Kool B, Katsumoto T, GirI V. A Case Report Of Hemophagocytic Lymphohistiocytosis (HLH) Associated with Anti-Pd-1 Immune Checkpoint Inhibitor (ICI) Therapy with Cemiplimab and Chronic Lymphocytic Leukemia (CLL) : Attempting Drug Removal with Plasma Exchange (PLEX) is not sufficient [Conference Abstract]. Pediatric Blood Cancer. 2023;70.

[48] Masood A, Wahab A, Clifford T, Weaver EJ, Ehsan H, El Ayass W (2021). Secondary hemophagocytic lymphohistiocytosis due to nivolumab/ipilimumab in a renal cell cancer patient-A case report. Clin Case Rep.

[49] Heynemann S, Vanguru V, Adelstein S, Kao S (2022). Hemophagocytic lymphohistiocytosis (HLH) and cytokine release syndrome (CRS) in a patient with oncogene-addicted metastatic non-small cell lung cancer (NSCLC) following combination chemotherapy-immunotherapy. Asia Pac J Clin Oncol.

[50] Martin-Kool B, Katsumoto T, GirI V. A Case Report of Hemophagocytic Lymphohistiocytosis (HLH) Associated with anti-PD-1 Immune Checkpoint Inhibitor (ICI) Therapy with Cemiplimab and Chronic Lymphocytic Leukemia (CLL) : Attempting Drug Removal With Plasma Exchange (PLEX) is not sufficient. Pediatric Blood Cancer. 2023;70 10.1002/pbc.30097

[51] Griffin G, Shenoi S, Hughes GC (2020). Hemophagocytic lymphohistiocytosis: An update on pathogenesis, diagnosis, and therapy. Best Pract Res Clin Rheumatol.

[52] Kubo T, Hirohashi Y, Tsukahara T, Kanaseki T, Murata K, Morita R, Torigoe T (2022). Immunopathological basis of immune-related adverse events induced by immune checkpoint blockade therapy. Immunol Med.

[53] Humblet-Baron S, Franckaert D, Dooley J, Bornschein S, Cauwe B, Schönefeldt S, Bossuyt X, Matthys P, Baron F, Wouters C (2016). IL-2 consumption by highly activated CD8 T cells induces regulatory T-cell dysfunction in patients with hemophagocytic lymphohistiocytosis. J Allergy Clin Immunol.

[54] Yin Q, Wu L, Han L, Zheng X, Tong R, Li L, Bai L, Bian Y (2023). Immune-related adverse events of immune checkpoint inhibitors: a review. Front Immunol.

[55] Lee DJ, Lee HJ, Farmer JR, Reynolds KL (2021). Mechanisms driving immune-related adverse events in cancer patients treated with immune checkpoint inhibitors. Curr Cardiol Rep.

[56] Johnson DB, Balko JM, Compton ML, Chalkias S, Gorham J, Xu Y, Hicks M, Puzanov I, Alexander MR, Bloomer TL (2016). Fulminant Myocarditis with Combination Immune Checkpoint Blockade. N Engl J Med.

[57] Gordon SR, Maute RL, Dulken BW, Hutter G, George BM, McCracken MN, Gupta R, Tsai JM, Sinha R, Corey D (2017). PD-1 expression by tumour-associated macrophages inhibits phagocytosis and tumour immunity. Nature.

[58] Jing Y, Yang J, Johnson DB, Moslehi JJ, Han L (2022). Harnessing big data to characterize immune-related adverse events. Nat Rev Clin Oncol.

[59] Gu T, Jiang A, Zhou C, Lin A, Cheng Q, Liu Z, Zhang J, Luo P (2023). Adverse reactions associated with immune checkpoint inhibitors and bevacizumab: a pharmacovigilance analysis. Int J Cancer.

[60] Bai S, Tian T, Pacheco JM, Tachihara M, Hu P, Zhang J (2021). Immune-related adverse event profile of combination treatment of PD-(L)1 checkpoint inhibitors and bevacizumab in non-small cell lung cancer patients: data from the FDA adverse event reporting system. Transl Lung Cancer Res.

[61] Yang J, Yan J, Liu B (2018). Targeting VEGF/VEGFR to Modulate Antitumor Immunity. Front Immunol.

[62] Toi Y, Sugawara S, Sugisaka J, Ono H, Kawashima Y, Aiba T, Kawana S, Saito R, Aso M, Tsurumi K (2019). Profiling Preexisting Antibodies in Patients Treated With Anti-PD-1 Therapy for Advanced Non-Small Cell Lung Cancer. JAMA Oncol.

[63] Sakakida T, Ishikawa T, Chihara Y, Harita S, Uchino J, Tabuchi Y, Komori S, Asai J, Narukawa T, Arai A (2020). Safety and efficacy of PD-1/PD-L1 blockade in patients with preexisting antinuclear antibodies. Clin Transl Oncol.

[64] Bagchi S, Yuan R, Engleman EG (2021). Immune checkpoint inhibitors for the treatment of cancer: clinical impact and mechanisms of response and resistance. Annu Rev Pathol.

[65] Leonardi GC, Gainor JF, Altan M, Kravets S, Dahlberg SE, Gedmintas L, Azimi R, Rizvi H, Riess JW, Hellmann MD (2018). Safety of programmed death-1 pathway inhibitors among patients with non-small-cell lung cancer and preexisting autoimmune disorders. J Clin Oncol.

[66] Arca M, Fardet L, Galicier L, Rivière S, Marzac C, Aumont C, Lambotte O, Coppo P (2015). Prognostic factors of early death in a cohort of 162 adult haemophagocytic syndrome: impact of triggering disease and early treatment with etoposide. Br J Haematol.

[67] Le RQ, Li L, Yuan W, Shord SS, Nie L, Habtemariam BA, Przepiorka D, Farrell AT, Pazdur R (2018). FDA approval summary: tocilizumab for treatment of chimeric antigen receptor T cell-induced severe or life-threatening cytokine release syndrome. Oncologist.

[68] Hailemichael Y, Johnson DH, Abdel-Wahab N, Foo WC, Bentebibel SE, Daher M, Haymaker C, Wani K, Saberian C, Ogata D (2022). Interleukin-6 blockade abrogates immunotherapy toxicity and promotes tumor immunity. Cancer Cell.

[69] Fujii T, Colen RR, Bilen MA, Hess KR, Hajjar J, Suarez-Almazor ME, Alshawa A, Hong DS, Tsimberidou A, Janku F (2018). Incidence of immune-related adverse events and its association with treatment outcomes: the MD Anderson Cancer Center experience. Invest New Drugs.

[70] Socinski MA, Jotte RM, Cappuzzo F, Nishio M, Mok TSK, Reck M, Finley GG, Kaul MD, Yu W, Paranthaman N (2023). Association of Immune-Related Adverse Events With Efficacy of Atezolizumab in Patients With Non-Small Cell Lung Cancer: Pooled Analyses of the Phase 3 IMpower130, IMpower132, and IMpower150 Randomized Clinical Trials. JAMA Oncol.

