# Supplementary material for: Hemophagocytic lymphohistiocytosis following pembrolizumab and bevacizumab combination therapy for cervical cancer: a case report and systematic review
Source: BMC Geriatr. 2024 Jan 8;24:32. doi: 10.1186/s12877-023-04625-3 (PMC10773023; doi:10.1186/s12877-023-04625-3)
Supplement: Supplementary file 1 — Additional file 1. [file 12877_2023_4625_MOESM1_ESM.pdf]

Supplementary table 1:

HLH-2004 diagnosis criteria
Fever ≥38.5 C
Splenomegaly
Cytopenia with at least two of the following (Hb <10 g/dL, Plt <100 000/ μL, Neu <1000/μL)
TG >265 mg/dL and/or fibrinogen <150 mg/dL
Ferritin >500 ng/mL
sIL-2R >age-adjusted laboratory-specific norms
Hemophagocytosis in bone marrow, spleen, lymph node, or liver
Low or absent NK cell activity

Note: Five of the eight criteria are needed to fulfill the HLH diagnosis.

Abbreviations: Hb, hemoglobin; Neu, neutrophils; NK, natural killer; Plt, platelets; sIL-2R, soluble interleukin-2 receptor; TG, triglycerides.

Supplementary table 2:

Table S2. The HScore

Parameter	No. of points (criteria for scoring)
Known underlying immunosuppression[a]	0 (no) or 18 (yes)
Temperature (°C)	0 (<38.4), 33 (38.4–39.4), or 49 (>39.4)
Organomegaly	0 (no), 23 (hepatomegaly or splenomegaly), or 38 (hepatomegaly and splenomegaly)
No. of cytopenias[b]	0 (1 lineage), 24 (2 lineages), or 34 (3 lineages)
Ferritin (ng/ml)	0 (<2,000), 35 (2,000–6,000), or 50 (>6,000)
Triglyceride (mmoles/liter)	0 (<1.5), 44 (1.5–4), or 64 (>4)
Fibrinogen (gm/liter)	0 (>2.5) or 30 (≤2.5)
Serum glutamic oxaloacetic transaminase (IU/liter)	0 (<30) or 19 (≥30)

Hemophagocytosis features on bone marrow aspirate	0 (no) or 35 (yes)

a Human immunodeficiency virus positive or receiving long-term immunosuppressive therapy (i.e., glucocorticoids, cyclosporine, azathioprine).
b Defined as a hemoglobin level of ≤9.2 gm/dl and/or a leukocyte count of ≤5,000/mm3 and/or a platelet count of ≤110,000/mm3.

Supplementary table 3:

Probability of hemophagocytic syndrome according to the HScore*

HScore	Probability of hemophagocytic syndrome, %
90	<1
100	1
110	3
120	5
130	9
140	16
150	25
160	40
170	54
180	70
190	80
200	88
210	93

HScore	Probability of hemophagocytic syndrome, %
220	96
230	98
240	99
250	>99

The best cutoff value for HScore was 169, corresponding to a sensitivity of 93%, a specificity of 86%, and accurate classification of 90% of the patients.
