# Supplementary material for: Hemophagocytic lymphohistiocytosis following pembrolizumab and bevacizumab combination therapy for cervical cancer: a case report and systematic review
Source: BMC Geriatr. 2024 Jan 8;24:32. doi: 10.1186/s12877-023-04625-3 (PMC10773023; doi:10.1186/s12877-023-04625-3)

Supplementary table 4:

PubMed search strategy.

#NO	Searches
1	“Programmed Cell Death 1 Receptor”[Mesh] OR “CTLA-4 Antigen”[Mesh]
2	“anti-PD-1”[Title/Abstract] OR “PD-1”[Title/Abstract] OR “anti-PD-L1”[Title/Abstract] OR “PD-L1”[Title/Abstract] OR “anti-PD(L)-1”[Title/Abstract] OR “PD(L)-1”[Title/Abstract] OR “CTLA-4”[Title/Abstract] OR “anti-CTLA-4”[Title/Abstract] OR ” anti-cytotoxic T-lymphocyte antigen-4”[Title/Abstract]
3	“nivolumab”[Title/Abstract] OR “pembrolizumab”[Title/Abstract] OR “atezolizumab”[Title/Abstract] OR “durvalumab”[Title/Abstract] OR “avelumab”[Title/Abstract] OR “ipilimumab”[Title/Abstract] OR “cemiplimab”[Title/Abstract]
4	“immune checkpoint inhibitor”[Title/Abstract] OR “immune checkpoint inhibitors”[Title/Abstract] OR “ICI”[Title/Abstract] OR “immune checkpoint blockade”[Title/Abstract] OR “ICB”[Title/Abstract]
5	‘Lymphohistiocytosis, Hemophagocytic’ (Mesh) OR ‘Hemophagocytic lymphohistiocytosis’ OR ‘Haemophagocytic lymphohistiocytosis’ OR ‘Hemophagocytic syndrome’ OR ‘Haemophagocytic syndrome’ OR ‘Macrophage Activation Syndrome’(Mesh)] OR ‘Macrophage Activation Syndrome’ OR MAS OR HLH OR hypercytokinaemia OR ‘cytokine storm’ OR ‘hemophagocytic activation syndrome’

6	(1 or 2 or 3 or 4)and 5 = 357

Supplementary table 5:

Embase search strategy.

#NO	Searches
1	"immune checkpoint inhibitor"/de OR "avelumab"/de OR "atezolizumab"/de OR "cemiplimab"/de OR "durvalumab"/de OR "ipilimumab"/de OR "nivolumab"/de OR "pembrolizumab"/de
2	atezolizumab:ti,ab,kw OR avelumab:ti,ab,kw OR cemiplimab:ti,ab,kw OR durvalumab:ti,ab,kw OR ipilimumab:ti,ab,kw OR nivolumab:ti,ab,kw OR pembrolizumab:ti,ab,kw
3	(("ctla-4" OR ctla4 OR "cytotoxic t-lymphocyte-associated protein 4" OR checkpoint OR "pd-1" OR pd1 OR "pd-l1" OR pdl1 OR "programmed death-ligand 1" OR "programmed cell death protein 1") NEAR/3 (block* OR inhibit*)):ti,ab,kw
4	"hemophagocytic syndrome"/exp OR "hemophagocytic syndrome"

5	("macrophage activati*" NEAR/2 syndrome*):ti,ab,kw
6	((erythrophagocyt* OR hemophagocyt*) NEAR/2 (histiocyt* OR hymphohistiocyt* OR lymphohistiocyt* OR reticulosis OR syndrome*)):ti,ab,kw
7	"cytokine storm"/exp
8	(1 or 2 or 3) and (5 or 6 or 7) = 1082

Supplementary figure 1:

.

Diagram of the PRISMA

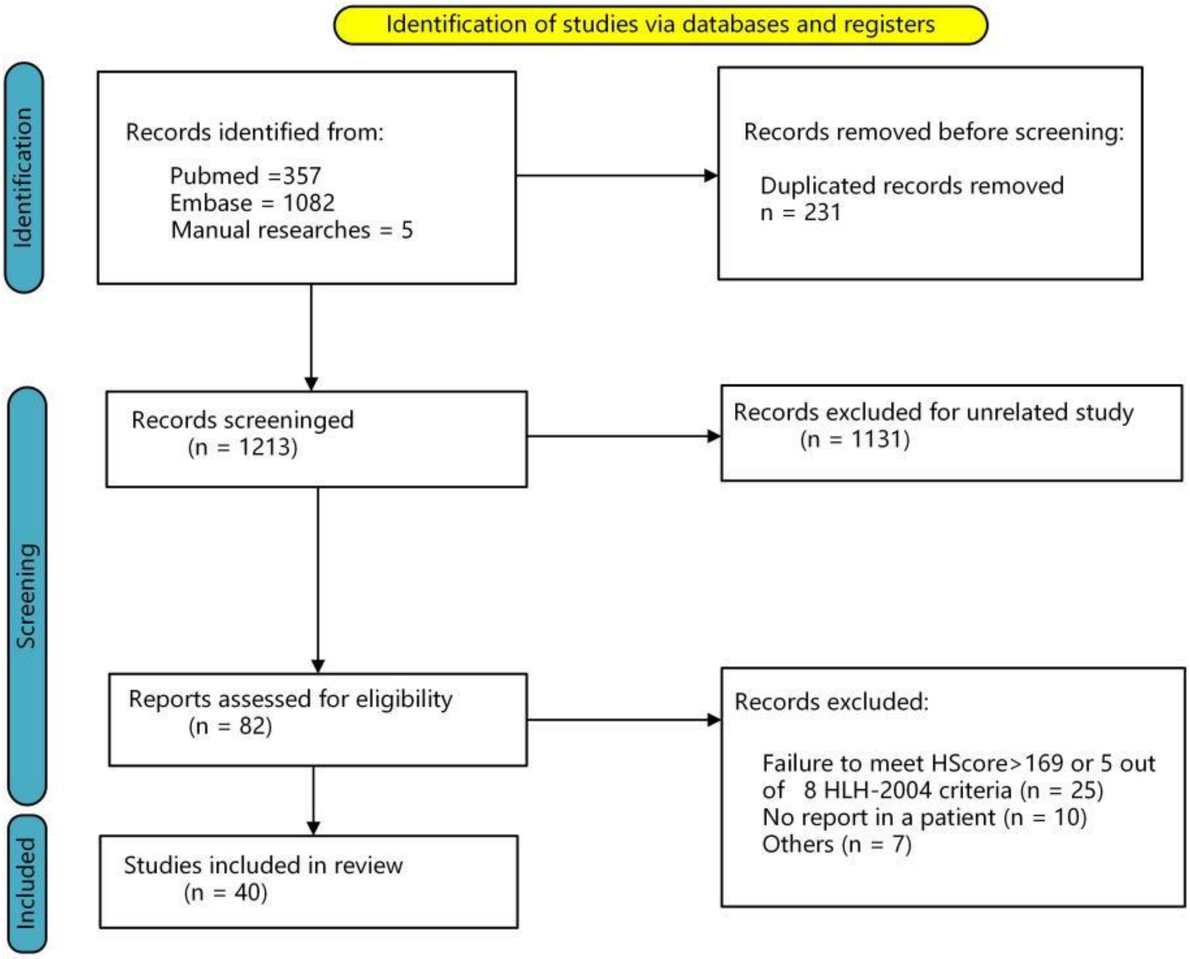

Supplement: Supplementary file 2 — Additional file 2. [file 12877_2023_4625_MOESM2_ESM.pdf]
