# Supplementary material for: Hemophagocytic lymphohistiocytosis following pembrolizumab and bevacizumab combination therapy for cervical cancer: a case report and systematic review
Source: BMC Geriatr. 2024 Jan 8;24:32. doi: 10.1186/s12877-023-04625-3 (PMC10773023; doi:10.1186/s12877-023-04625-3)
Supplement: Supplementary file 3 — Additional file 3. [file 12877_2023_4625_MOESM3_ESM.pdf]

Naranjo Adverse Drug Reaction Probability Scale for Pembrolizumab and Bevacizumab

Question	Answer Options	Scores	pembrolizumab	bevacizumab
Are there previous conclusive reports on this reaction?	Yes / No / Do not know or not done	+1 / 0 / 0	1	0
Did the adverse events appear after the suspected drug was given?	Yes / No / Do not know or not done	+2 / -1 / 0	2	2
Did the adverse reaction improve when the drug was discontinued or a specific antagonist was given?	Yes / No / Do not know or not done	+1 / 0 / 0	1	0
Did the adverse reaction appear when the drug was readministered?	Yes / No / Do not know or not done	+2 / -1 / 0	0	0
Are there alternative causes that could have caused the reaction?	Yes / No / Do not know or not done	-1 / +2 / 0	-1	-1
Did the reaction reappear when a placebo was given?	Yes / No / Do not know or not done	-1 / +1 / 0	1	1
Was the drug detected in any body fluid in toxic concentrations?	Yes / No / Do not know or not done	+1 / 0 / 0	1	1
Was the reaction more severe when the dose was increased, or less severe when the dose was decreased?	Yes / No / Do not know or not done	+1 / 0 / 0	0	0
Did the patient have a similar reaction to the same or similar drugs in any previous exposure?	Yes / No / Do not know or not done	+1 / 0 / 0	0	0
Was the adverse event confirmed by any objective evidence?	Yes / No / Do not know or not done	+1 / 0 / 0	1	0
Total Score			6	3

Scoring: ≥ 9 = Definite ADR (Adverse Drug Reaction), 5-8 = Probable ADR, 1-4 = Possible ADR, 0 = Doubtful ADR
